# “The NET Outcome”: Are Neutrophil Extracellular Traps of Any Relevance to the Pathophysiology of Autoimmune Disorders in Childhood?

**DOI:** 10.3389/fped.2016.00097

**Published:** 2016-09-13

**Authors:** Stavros Giaglis, Sinuhe Hahn, Paul Hasler

**Affiliations:** ^1^Laboratory of Prenantal Medicine, Department of Biomedicine, University Clinics Basel, Basel, Switzerland; ^2^Department of Rheumatology, Kantonsspital Aarau, Aarau, Switzerland; ^3^University Women’s Hospital, University Clinics Basel, Basel, Switzerland

**Keywords:** neutrophils, NETs, NETosis, biomarkers, juvenile idiopathic arthritis, SLE

## Abstract

Neutrophil extracellular trap (NET) formation represents a form of cell death distinct from apoptosis or necrosis, by which invading pathogens are simultaneously entangled and potentially eliminated. Increased NET formation is observed in systemic lupus erythematosus (SLE), rheumatoid arthritis, antineutrophil cytoplasmic antibody-associated small vessel vasculitis, antiphospholipid antibody syndrome (APS), and psoriasis. NETs contribute to the pathogenesis of autoimmunity by exposing cryptic autoepitopes, which may facilitate the generation of autoantibodies, induce the production of interferons, and activate the complement cascade. In SLE, augmented disease activity and renal disease are associated with increased NET formation, so that NETs could serve as a marker for the monitoring of disease activity. NETs can additionally cause endothelial cell damage and death and stimulate inflammation in atheromatous plaques, adding to the accelerated atherosclerosis witnessed in autoimmune disease. Since NETs induce production of interferons, assessing the extent of NET formation might facilitate the prediction of IFN-alpha levels and identification of SLE patients with presumably better responses to anti-IFN-alpha therapies or other novel therapeutic concepts, such as *N*-acetyl-cysteine and inhibitors of DNase 1 and peptidylarginine deiminase 4 (PAD4), which also target NETs. In summary, the study of NETs provides a novel approach to the understanding of autoimmune disease pathogenesis in childhood and opens new vistas in the development of sensitive disease markers and targeted therapies.

## Introduction

Neutrophils play a key role in innate immunity with multiple strategies for defending the host against pathogens. Neutrophils initially migrate to the site of infection and exert their critical roles through phagocytosis, degranulation and secretion of antimicrobial factors, production of reactive oxygen species (ROS), and release of neutrophil extracellular traps (NETs) (1–3). Although the primary function of neutrophils is to protect the body from hazardous microbes, the influx of neutrophils to an inflamed site can contribute directly to tissue damage and the progression to chronic inflammation. Neutrophils mediate a pro-inflammatory response to tissue injury or infection by releasing cytokines that recruit and stimulate other immune cells and promote the activation of adaptive immunity. Neutrophil granulocytes have been traditionally viewed as short-lived cells that die at sites of inflammation; increasing evidence, however, suggests that they can expand their life span upon specific stimuli and transmigrate away from inflammatory loci (2–4).

**Figure 1:**
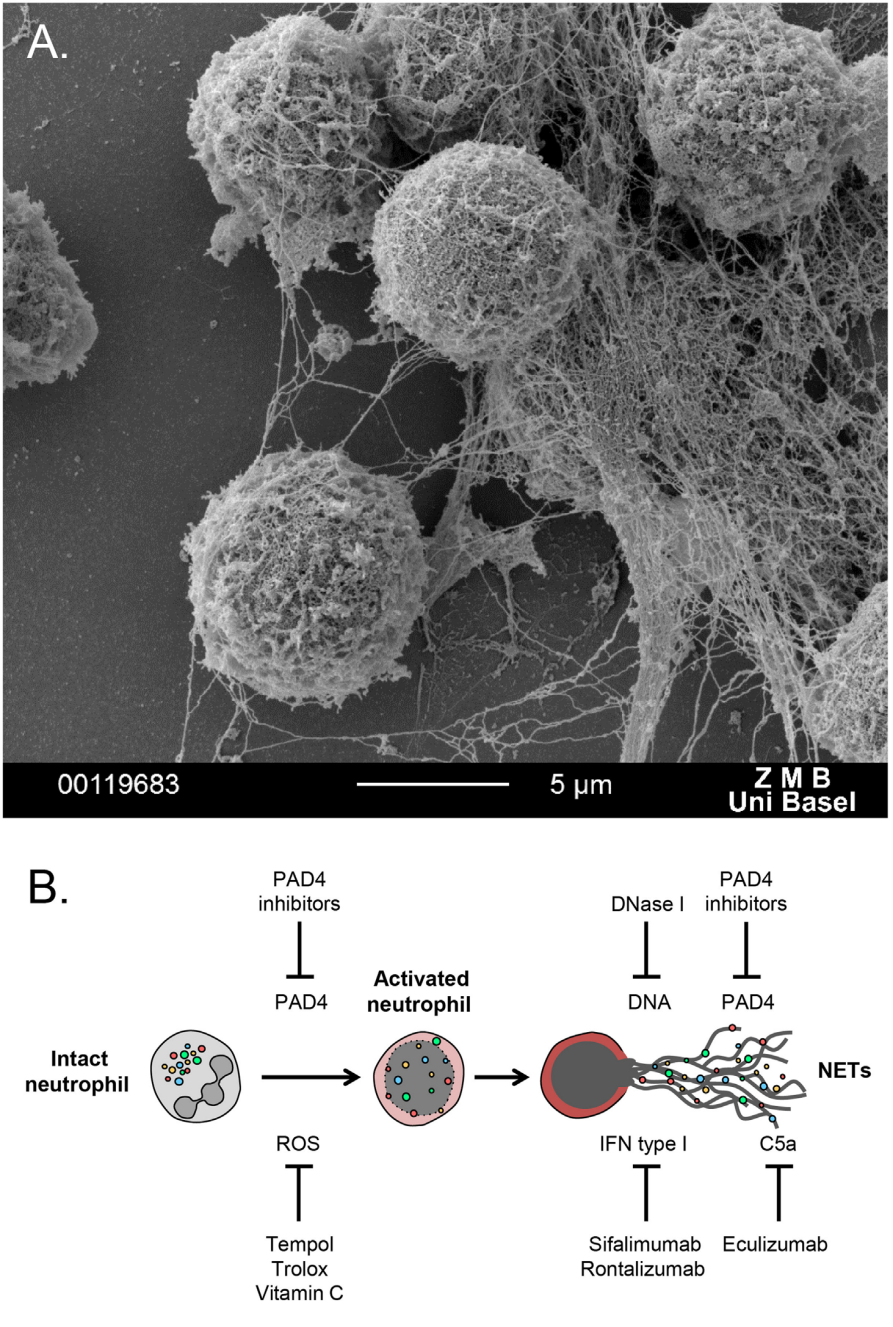
**(A)** Scanning electron microscopy image of polymorphonuclear neutrophils undergoing NETosis, after *in vitro* treatment with PMA for 3 h. **(B)** Schematic overview of potential NETs targeting therapies. A series of antioxidants can reduce generation of ROS, which triggers NET formation. DNase 1 digests extracellular DNA and, thus, degrades NETs. PAD4 inhibitors block histone citrullination, an important event for autoantibody generation in autoimmune disease. Sifalimumab and rontalizumab are monoclonal antibodies to IFN-a, which is a byproduct of NETosis that can further stimulate neutrophil activation and NET release. Eculizumab, a monoclonal antibody to C5, can block complement activation that stimulates formation of NET.

More recently, neutrophils were found to form extracellular meshes called NETs, which are composed mainly of DNA, histones, and granular proteases (Figure 1A). NETs were first observed as a unique form of cell death, distinct from apoptosis or necrosis (5), a process further described as NETosis (6, 7). NETs trap and restrain invading pathogens and utilize their highly localized focus of antimicrobial granular peptides to degrade virulent factors and even kill microorganisms (6–8).

Neutrophil extracellular traps were originally defined being sparked by stimulation with Gram-positive and -negative bacteria, fungi, and parasites, together with agents as IL-8, lipopolysaccharide (LPS), and PMA (6, 7). The most prominent pathway mediating NET formation appears to be NADPH oxidase-generated ROS, with the granular enzymes myeloperoxidase (MPO) and neutrophil elastase (NE) promoting chromatin decondensation and peptidyl arginine deiminase (PAD4) catalyzing histone citrullination (9–12). Moreover, recent data show that NET formation is directly linked to autophagy (13), while regulation of autophagy is closely tied to ROS (14). Most importantly, ROS levels determine whether the autophagy response ends in NETosis (13). The precise ways, however, in which ROS interfere with the signaling network behind NETs and autophagy are only partly understood.

The identification of NETs has been accomplished *via* microscopic techniques, such as immunofluorescence microscopy, transmission electron microscopy (TEM), and scanning electron microscopy (SEM). Recently, a series of novel immunocyto- and histochemical procedures allowed improved imaging and quantification of NETs. On the one side, *in vitro* live-cell imaging led to profound new concepts over the mechanisms of NET generation. These include fluorescence microscopy and spectroscopy (6, 15), electron microscopy (15, 16), and lately flow cytometry with simultaneous imaging (17, 18). On the other side, diverse intravital, *in vivo*, and *in situ* microscopic techniques led to deeper insights into the role of NET formation during health and disease (12, 19).

Moreover, the deposition of NETs observed in various inflammatory pathologies was associated with the circulating cell-free DNA (cfDNA) levels in biological fluids of the respective patients, such as plasma and serum (15, 20, 21). Therefore, circulatory cfDNA could eventually be utilized as a surrogate marker of NETs in these pathologies, while determination of the DNA levels might facilitate monitoring the disease activity and assessment of the effectiveness of a selected therapeutic strategy.

Although they play a key role in the defense against pathogens, NETs may cause undesirable effects to the host. Most importantly, there has been increased interest in the role of neutrophils and NETs in autoimmunity. Augmented NET formation was first described in preeclampsia and ANCA-associated vasculitis, and successively in a series of autoimmune conditions, including psoriasis, SLE, antiphospholipid antibody syndrome (APS), and rheumatoid arthritis (RA) (15, 22–26) (Table 1).

**Table 1:** **Involvement of neutrophils and NETs in autoimmune diseases during childhood and adulthood**.

Disease	Proposed role of neutrophils and/or NETs	Reference
**Childhood**		
Juvenile idiopathic arthritis	NETs as a source of citrullinated pathogenic autoantigens Involvement of the S100A proteins originating from activated neutrophils in the local tissue damage	(46–50)
Sytemic lupus erythematosus	dsDNA extruded as an pathogenic immunostimulatory autoepitope, promoting IFNα production – LDGs Decreased NET degradation capacity Endothelial cell damage and dysfunction	(26, 41, 52–55)
Spondyloarthritis	Active IL-12/IL-23 and the IL-17 cytokine pathways in MPO+/CD15+ neutrophils	(56–58)
Juvenile dermatomyositis	Abnormal generation of NETs could be a factor initiating and exaggerating the lung involvement Decreased NET degradation capacity	(59)
Type 1 diabetes mellitus	Involvement of the granular proteolytic enzymes NE and MPO originating from NETs affect the IRS-1 dependent glucose tolerance and local tissue damage	(64–67)
**Adulthood**		
Rheumatoid arthritis	NETs as a source of citrullinated pathogenic autoantigens	(15, 23)
Psoriasis	LL-37 extruded with the NETs drives production of IFNα	(26)
ANCA-associated vasculitis	The granule protein contents of NET may be the antigen source for pathogenic ANCAs Increased NET formation may contribute to clot formation	(22)
APS	NETs could promote clot formation	(25)

Neutrophil extracellular trap are an obvious source of nuclear material. Among these are a range of cytoplasmic and extracellular citrullinated antigens, well-established targets of anticitrullinated protein antibodies (ACPAs) found in RA (15, 23). Not only do the protein contents of NETs serve as the targets for autoantibody and immune complex formation but they also induce further NETosis, resulting in a harmful positive-feedback loop. These factors form an inflammatory microenvironment that may trigger a strong autoimmune response in individuals with the corresponding susceptibility (27, 28).

Pro-inflammatory cytokines, such as tumor necrosis factor α (TNFα) and interleukin-17 (IL-17), as well as autoantibodies stimulate the formation of NETs and also affect their protein composition (23). Additionally, NETs have been shown to stimulate autoimmunity *via* the production of interferons and activation of the complement cascade. Antimicrobial granular proteolytic enzymes and the DNA contents of NETs induce plasmacytoid dendritic cell (pDC) interferon α (IFNα) production in SLE and psoriasis, as well as in the case of invading viruses (24). Interferons activate both innate and adaptive immune systems, inducing a Th1 immune response and stimulating B cells toward the generation of autoantibodies.

In addition to the elevated NET formation in patients with autoimmune diseases, increased NET stability and defects in the clearance of NETs have also been observed, particularly in SLE and ANCA-associated vasculitis. In a subset of SLE and ANCA-associated vasculitis patients, a decreased capacity for NET clearance due low DNase 1 activity as well as the presence of DNase 1 inhibitors has been described. Moreover, antibodies may prevent DNase 1 access to NETs, thus protecting them from degradation (25, 29–31). Finally, complement appears to directly interact with NETs and inhibit DNase 1-mediated NET degradation, while non-degraded NETs activate complement *in vitro* (32). Taken together, a detrimental positive-feedback loop seems to activate complement by the formation of NETs, which, in turn, increases NETs even further by preventing their degradation.

Neutrophil extracellular trap represent a relatively novel therapeutic target in autoimmune disease. There are several key events throughout neutrophil activation toward NETosis, which could be aimed to inhibit NET formation (Figure 1B). Given the involvement of ROS and oxidative stress in NETosis and autoimmunity, the utilization of antioxidants, such as Trolox, Tempol (33), and vitamin C (34), which prevent NET release, could be introduced for treating autoimmune inflammatory diseases. Another target for blocking NETosis is PAD4, inhibitors of which might ameliorate disease manifestations but also improve endothelial dysfunction (35). Recombinant DNase 1 could potentially degrade NETs, leading to efficient clearance of harmful debris. Finally, a series of recombinant human monoclonal antibodies are shown to restrain NET formation, acting indirectly on molecules released from the activated neutrophils together with NETs. These recombinant mAbs could be administered for neutralization of the cytokine byproducts of the NETotic process, such as IFN and members of the complement system (36, 37).

## Autoimmune Conditions in Childhood

The role of neutrophils and NETs in pediatric rheumatology is currently gaining increasing attention. Childhood rheumatic disorders are categorized into autoimmune and autoinflammatory conditions (38). Neutrophil infiltration into the tissues is present in both autoinflammatory diseases characterized by activation of the innate immune system, and classic autoimmune diseases are characterized by the presence of autoantibodies and autoreactive T cells. This makes the common basic effector pathways evident. In the pediatric setting, special attention must be given to the possibility of congenital immune deficiencies that present with features of autoimmune disease. For instance, children may develop manifestations of inflammatory bowel disease (IBD) with neutrophilic inflammation due to primary genetic defects in the innate immune system, including chronic granulomatous disease (CGD) with mutations in NADPH oxidase (39).

### Neutrophils and NETs in JIA

Juvenile idiopathic arthritis (JIA) is the most common chronic autoimmune disease of childhood (40). JIA represents a group of entities that start prior to puberty with incident arthritis lasting at least 6 weeks without any additionally identified cause (41). The 2001 ILAR classification criteria divide JIA into several disease categories based on the number and type of affected joints and the presence of systemic presentations (42). Although different JIA categories share common pathogenic mechanisms and therapeutic strategies, they are distinct entities, and the role of neutrophils in pathogenesis likely differs by JIA subtype as suggested by both heterogeneous clinical characteristics and underlying genetics.

A series of factors indicate that oligoarticular and rheumatoid factor (RF)-negative polyarticular JIA are typical autoimmune diseases, including the linkage with specific HLA haplotypes, as well as the presence in the synovial fluid of specific memory Th1 lymphocytes (43, 44). Genome wide association studies have detected associations with a series of loci that overlap with classic autoimmune diseases, including adult RA, multiple sclerosis (MS), and type I diabetes mellitus (T1D) (43, 45).

Gene expression analyses in neutrophils from patients suffering from RF-negative polyarticular JIA revealed modulation of IL-8- and IFNγ-regulated genes (46). Alleles related to calcium flux and ROS generation have been reported in affected neutrophils (47). These abnormalities distinguished JIA from healthy controls, but not active from inactive disease, suggesting the existence of additional intrinsic cellular defects. Furthermore, there is evidence for chronic activation of peripheral neutrophils in pediatric patients with polyarticular disease like in adult RA (46).

S100A proteins are zinc and calcium-binding proteins that represent up to half of the total cytosolic protein in phagocytes. Neutrophils and monocytes produce the S100A8/S100A9 heterodimer, also known as calprotectin, while S100A12 is primarily produced by activated neutrophils. Upon binding the receptors for advanced glycation end products (RAGE), S100A proteins activate endothelial cells, inducing expression of adhesion molecules and chemoattractants, thus increasing leukocyte recruitment (48). Calprotectin also triggers toll-like receptor 4 (TLR4), promoting secretion of pro-inflammatory cytokines, such as IL-6, IL-1β, and TNFα (49). Levels of calprotectin and S10012A rise to very high levels within inflamed joints, but increased serum levels have been also reported, reflecting a state of intense neutrophil activation (39).

Interestingly, calprotectin was found to be the major antifungal component in NETs. Absence of calprotectin resulted in the complete loss of antifungal activity *in vitro* and *in vivo* (50). Moreover, reconstituted NET formation after gene therapy for human CGD was associated with rapid resolution of pulmonary aspergillosis *in vivo*, underlining the role of functional NADPH oxidase in NET formation and calprotectin release for antifungal activity (51). The circulating levels of S100A seem to have an even higher impact in systemic JIA, in which neutrophils are the primary effector cells. Clinical symptoms, such as fever, rashes, hepatosplenomegaly and serositis, and the underlying genetic background of the affected individuals, place this condition firmly into the “autoinflammatory disease” category, with IL-1 and IL-6 playing major roles in the manifestations of this disorder (44).

Although clearly implicated in JIA, the influence of neutrophils and NETs on pathogenesis and the utility of biomarkers for neutrophil activity require further studies.

### Neutrophils and NETs in SLE

Systemic lupus erythematosus (SLE) is a systemic autoimmune disease with multi-organ involvement characterized by autoantibody generation and deposition of immune complexes in the tissues. The main emphasis in SLE pathogenesis has been laid on the adaptive immune system. Nevertheless, recent studies have brought attention to the role of the innate immune arm of defense, and neutrophils in particular (41).

In contrast to arthritis, where neutrophils are considered to be generally primed, even in the circulation (15, 23), there is evidence that neutrophils from SLE patients show compromised activation and reduced capacities for oxidative burst, chemotaxis, adhesion, and phagocytosis (26, 41). Lupus neutrophils, however, might present an activated phenotype locally in the vasculature, where they contribute to the development of vasculitis by damaging the endothelium (26, 41, 52). A possible reason is most likely the presence of a special neutrophil subset in the circulation, determined by a distinctive gene expression profile, termed low-density neutrophils (LDGs). Although deficient in some typical neutrophil effector functions (degranulation, phagocytosis), LDGs exert a pro-inflammatory phenotype as measured by cytokine release (including type I IFNs) and enhanced capacity for endothelial damage (53). Compared with normal density neutrophils, LDGs have an unusual tendency toward NET formation in the absence of any secondary stimuli and externalize various immunostimulatory proteins (53). LDGs show an enhanced capacity to stimulate pDCs to secrete type I interferon, a major cytokine implicated in lupus (24, 54). Moreover, NETs are a possible source of autoepitopes in lupus (41). Otherwise, neutrophils from pediatric SLE patients exert increased apoptosis that could also act as a source of autoantigens. In particular, serum from pediatric lupus patients induces neutrophil apoptosis, which is abrogated by granulocyte-macrophage colony-stimulating factor (GM-CSF) treatment, suggesting a potential therapeutic benefit of GM-CSF in SLE patients (55).

In conclusion, most of the current evidence supports a role of neutrophils in the pathogenesis of this disease, particulary by LDGs.

### Neutrophils and NETs in Spondyloarthritis

Calprotectin levels are found increased in enthesitis-related arthritis (ERA). These elevations, though, are less than in other types of juvenile arthritis, suggesting a reduced role for neutrophils in spondyloarthritis (56). Recent studies underline the significance of the IL-12/IL-23 and the IL-17 cytokine pathways in the pathogenesis of the disease (57). Remarkably, MPO-positive CD15-positive neutrophils were foremost in producing IL-17, suggesting an unexpected role for the innate immune system (58). In this context, cytokine production may be the main driver of neutrophil mediated inflammation rather than NET formation.

### Neutrophils and NETs in Juvenile Dermatomyositis

The pathogenesis of the systemic autoimmune disorders dermatomyositis (DM) and polymyositis (PM) remains unclear. Patients suffering from DM and PM were shown to be exposed to large amounts of NETs (59). Compared to control subjects, these patients exhibited a significantly enhanced capacity for inducing NETs, which was supported by elevated levels of plasma LL-37 and cfDNA in the circulation (59). More importantly, DM and PM patients did not show an adequate ability to clear NETs sufficiently due to the low activity of DNase 1.

Moreover, patients suffering from DM and PM presenting with interstitial lung disease exhibited the lowest NETs degradation capacity *in vitro*. DNase 1 activity in patients with disease-specific autoantibodies was significantly lower than in patients without, while treatment with glucocorticoids seemed to improve this condition (59). This finding suggests that abnormal regulation of NETs may be involved in the disease pathogenesis and could be one of the factors that initiate and exaggerate lung involvement.

### Neutrophils and NETs in Type 1 Diabetes Mellitus

Metabolic diseases represent a leading health challenge (60). Microvascular complications, including diabetic nephropathy, retinopathy, and neuropathy, are major causes of morbidity and mortality in individuals suffering from diabetes worldwide. Current therapies are still inadequate, as high glucose levels promote activation, dysfunction, and apoptosis of both intravascular and extravascular immune cells (61).

Several recent studies revealed unexpected roles for neutrophils in metabolic conditions. It has long been acknowledged that obesity, a major cause of type 2 diabetes mellitus (T2D) and metabolic syndrome broadly, exhibits a distinct pro-inflammatory character that contributes to insulin resistance (62). Neutrophils are present in increased numbers in obese patients and migrate into the adipose tissue (63). Moreover, a strong increase of neutrophil numbers in the adipose tissue, accompanied by elevated release of NE and high NE activity is confirmed (64). Most importantly, inhibition of NE lead to a remarkable improvement of glucose tolerance and insulin sensitivity *in vivo*. NE degrades insulin receptor substrate 1 (IRS-1) in hepatocytes, inhibiting the insulin-driven signal transduction, akin to a mechanism proposed previously in lung cancer cells (65).

Furthermore, a role for neutrophils in the pathogenesis of autoimmune type 1 diabetes (T1D) was implied, which is usually diagnosed in children and young adults, previously known as juvenile diabetes. Neutrophils are involved by the triggering of pDC cytokine production and subsequent activation of the adaptive immune system (66). This study, among others, placed neutrophils high in the rank of possible players involved in the pathogenesis of autoimmunity, adding a crucial influence in shaping the adaptive immune response (15, 27, 54). In this context, a recent report confirmed a clear elevation of NE and proteinase 3 (PR3) in T1D patients, which was associated with elevated formation of NETs (21). These findings support a role of neutrophil activation and augmented protease activities in the early pathogenesis of β-cell autoimmunity and also suggest that circulating NE and PR3 could serve as sensitive diagnostic markers for T1D (21).

Moreover, NETs were recently shown to impair wound healing, particularly in diabetes, where neutrophils are more susceptible to form NETs. Inhibition of NET formation or cleaving NETs with DNase 1 was shown to improve wound healing and reduce NET-driven chronic diabetic inflammation (67). Finally, high glucose and hyperglycemia increase NET release and circulating markers of NETosis, respectively, providing a link between neutrophils, inflammation, and tissue damage in T2D (68), while MPO inhibition is the suggested potential strategy for prevention and treatment of insulin resistance (69).

## Conclusion

The study of neutrophils and NETs offer insight into the pathogenesis of multiple autoimmune diseases, since they are of high relevance in a broad series of such disorders. There are yet only few studies reporting on the role of neutrophils in pediatric autoimmunity; however, there is currently growing interest in relating the findings in adult disease to pediatrics.

Although neutrophils are the most common cells in the synovial fluid during arthritis, the understanding of their role in pathogenesis is only emerging. Studies of neutrophils in oligo- and polyarticular JIA reveal intrinsic activity-independent abnormalities in gene expression. Evidence of activation of circulating neutrophils, such as S100A proteins and surrogate markers for NET formation, appear as promising biomarkers for overt and subclinical disease activity. Nevertheless, prospective studies regarding the practical application of these neutrophil-related biomarkers are required.

Distinct activated neutrophil subsets and altered apoptosis have been described in lupus. NETs may be the source of key autoantigens and potent inducers of IFNα. The importance of the IL-12/IL-23 and IL-17 cytokine pathways in the neutrophil-mediated pathogenesis of spondyloarthritis were recently implied.

Dermatomyositis is currently considered as a NET-related pathology due to the detection of overt NET formation and the reduced NETs clearance capacity.

Moreover, in type 1 diabetes mellitus, activated neutrophils contribute to the pathogenetic process, especially *via* the granular proteolytic enzymes NE and MPO, which may originate from enhanced NET formation.

In summary, neutrophil NETs are shown to be of high relevance in the pathobiology of a series of childhood autoimmune disorders and provide the potential for developing successful disease markers and ultimately novel targeted effective therapeutics, such as DNase 1, PAD inhibitors, and anti-IFN therapies. Clarifying the remaining issues will pose novel implications for a broad range of pediatric rheumatologic conditions’ diagnosis and treatment.

## Author Contributions

SG devised and wrote the manuscript. SH and PH critically reviewed the manuscript.

## Conflict of Interest Statement

The authors declare that the research was conducted in the absence of any commercial or financial relationships that could be construed as a potential conflict of interest.
